# The maintenance of regional dialects: a matter of gender? Boys, but not girls, use local varieties in relation to their friends' nativeness and local identity

**DOI:** 10.3389/fpsyg.2014.01251

**Published:** 2014-10-31

**Authors:** Stéphanie Barbu, Nathael Martin, Jean-Pierre Chevrot

**Affiliations:** ^1^Laboratory EthoS – Animal and Human Ethology, UMR 6552-CNRS, University of Rennes 1Rennes, France; ^2^Laboratory LIDILEM – Linguistique et Didactique des Langues Etrangères et Maternelles, University of Grenoble AlpesGrenoble, France; ^3^IUF – Institut Universitaire de FranceParis, France

**Keywords:** regional dialect, gender, social networks, friendships, peers, convergence, accommodation, children

## Abstract

The linguistic diversity enduring beyond institutional pressures and social prejudices against non-standard dialects questions the social forces influencing language maintenance across generations and how children contribute to this process. Children encounter multi-dialectal interactions in their early environment, and increasing evidence shows that the acquisition of sociolinguistic variation is not a side issue but an inherent part of the general acquisition process. Despite these recent advances in sociolinguistic acquisition, children's sociolinguistic uses remain under-studied in relation to peer social networks and the ability to use dialect for identity purposes. Our study focused on a grammatical sociolinguistic variable consisting of the alternation between a regional and a standard variant of the third person object pronoun in French. The regional variant is a remnant of the Francoprovençal language and its usage by adults is strongly associated with local identity in the French Alps. We described, using questionnaires, the social networks of 117 10–11 year-old girls and boys living in the same restricted rural area. Thirteen native target children (7 girls and 6 boys) were selected from the sample, as well as 39 same-sex friends chosen according to their place of birth (native vs. non-native) and the duration of their friendship with the targets (number of years they have known each other). The target children were recorded during spontaneous dyadic conversations during free play at school with each category of friends. Target boys, but not girls, used the regional variant significantly more frequently with their long-term native friends than with their non-native friends. This adjustment mirrored their partners' uses. Moreover, with long-term native friends, boys used the regional variant twice as frequently as girls. Boys appeared thus as key actors in the maintenance and the diffusion of regional cues in local social networks.

## Introduction

In a number of modern industrialized countries, as in Europe, some regional languages tend to disappear or have already disappeared (Hornsby and Agarin, 2012). This is the case for the Francoprovençal language that extends over adjoining geographical areas of France, Switzerland and Italy. This regional language is less and less spoken in Italy and in France; even though it benefits from more vivacity in its Italian area. Moreover, regarding the French area, for several decades now the language is no longer passed from one generation to the next (Martin, 2011); but endangered languages can resist standardization in many ways. One of them is the use of linguistic cues from regional languages in regional varieties of national languages.

The linguistic diversity enduring beyond institutional pressures and social prejudices against non-standard dialects (i.e., through national language policies, school, media, as well as stigmatization and negative social judgments) questions the social forces underlying dialect maintenance across generations and how children contribute to this process (Chambers and Trudgill, 1998; Chambers, 2003). In adults, Holmquist's study (1985) in a rural village in the Spanish Pyrenees showed that it is men, especially those engaged in traditional mountain agriculture (vs. more modern farming), who employ more the regional vocalic forms typical of the local rural dialect. However, the role of the different age groups in this indirect process of language maintenance is not clearly understood. The use of vernacular forms within peer groups has been documented in Italy where young people insert expressions from regional dialects into their speech, both for fun and for pragmatic reasons (Radtke, 1993), suggesting that peer groups may play a role in the process of regional dialect maintenance.

Most humans carry regional and social markers from their native community. These markers have a local adaptability, namely significance for local identity and relationships, within local social networks in particular (Chambers, 2003). A number of studies have investigated the relationship between language uses and social network structure in adults and adolescents. These studies all point in the same direction: the more individuals are integrated in their local social network, the more they show the typical uses of their community or their peer group (see Labov, 2001; Milroy, 2002 for reviews). This influence of social networks has been evidenced at various linguistic levels, from sounds to syntactic constructions, in a variety of communities, both urban and rural.

In urban communities, Milroy (1980) seeking in the neighborhood of Belfast in Northern Ireland, what makes working class speech local, found a relationship between the use of local vernacular varieties and the density (i.e., contacts are interconnected) and multiplexity (i.e., interacting with the same people in a variety of contexts) of men's working class social networks. Labov (2001) provided a similar account for Philadelphia suburban neighborhoods. Similar trends have been evidenced in adolescents: both working class British boys (11–16 years) recorded with friends in Reading playgrounds (Cheshire, 1997, 2009) or underprivileged Afro-American boys (12–17 years) in youth gangs in South Harlem (Labov, 1972a, 2001), use non-standard features at frequencies related to the boys' participation in their local peer group culture. Peer group membership of high school students in Detroit suburbs appeared clearly in their differential uses of linguistic resources (Eckert, 2000, 2012): Just as they use clothes or hairstyles, adolescents use different language styles to enact peer group identity.

These social trajectories were also observed in small rural communities where the local regional dialect competed with the standard linguistic varieties, as evidenced by Lippi-Green (1989) in an isolated Austrian village: the higher the level of integration of speakers within local social networks, the higher their use of vernacular phonological varieties. Beaulieu and Cichocki (2002) observed a similar phenomenon for morphosyntactic variables in an Acadian French community in a small fishing village (in northeast New Brunswick, Canada) where the social network was the best predictor of non-standard uses, well beyond speakers' sociodemographic characteristics. It can be noticed that cited network studies often relied on small samples. Network and peer group integration effects did not systematically arise over and above socioeconomic variables when larger samples were concerned such as in Labov's work in Philadelphia (Labov, 2001).

Taken together, these studies conducted with adults or adolescents in various linguistic and social settings present nevertheless convergent evidence that speakers with strong ties within the local community maintain their local dialects more vigorously than others (Labov, 2001; Milroy, 2002). Close-knit networks appear thus as an important mechanism in dialect maintenance. These studies also revealed that, at least in some communities and for some linguistic variables, uses of the local dialect are age-graded and gendered, the oldest men showing the highest uses of vernacular varieties, suggesting a different role of age groups across generations and genders in dialect conservatism (Lippi-Green, 1989; Beaulieu and Cichocki, 2002). This leads us to question the contribution of younger speakers as children's dialectal uses remain under-studied in relation to peer social networks and the ability to use dialect for identity purposes.

Children encounter multi-dialectal interactions in their early environment, and increasing evidence shows that the acquisition of sociolinguistic variation is not a side issue but an inherent part of the general acquisition process taking place first in the family (Roberts, 2002; Foulkes and Docherty, 2006; Nardy et al., 2013 for reviews). Input from primary caregivers is crucial in this process, at least during early childhood. Indeed, strong correlations were found between caregivers' uses of non-standard varieties and children's uses under 4 years old (for instance in a Scottish dialect: Smith et al., 2007, 2013). However, as children grow up, language socialization spreads across various interactional settings, at school with peers in particular. By studying non-migrant children and adolescents between 6 and 18 years old in a rural Syrian village, Habib (2014) documented a shift from urban varieties, acquired first from out-of-town mothers, to local rural varieties starting at age 9. This shift was not related to peers' influences *per se*, but merely to an increasing awareness of the social meaning of the local rural varieties and an active construction of local and gender identity in the group of 9–11 year-olds, especially for boys.

Empirical evidence of the influence of peer group has been provided by studies of speakers moving to a new dialectal area and children's subsequent second dialect acquisition. In a number of cases, children do not speak like their parents, but instead follow the patterns of their peers (Chambers, 2002). As described above for adults and adolescents, a child's integration into the local peer group is crucial in determining whether she or he adopts the local dialect feature (e.g., Labov, 1972b; Kerswill and Williams, 2000). Although Payne (1980) reported that the major social variable was age of arrival in the community, Labov's re-analysis of Payne's data yielded a different result: the density of the speaker's social network appeared as the most significant independent variable (above effect of age, age of arrival, or years spent in the community). The influence of other children and peers outside the family has been more rarely studied in first dialect acquisition. Nevertheless, one study investigated the relationship between children's uses and peer social network showing that the preschool children who interact more frequently in the classroom adopt similar uses of non-standard varieties of French variables leading to a convergence in children's uses over a school year (Nardy et al., in press). Thus, peer social interactions influence children's dialectal uses at an early age and appear as an important piece of the puzzle to understand the social dynamics of dialect maintenance or change.

Although family plays a primary role in first dialect acquisition and transmission, children also go to school at an early age and there they are in extended contact with peers potentially from other social or geographical backgrounds. This leads us to question what children are doing socially with linguistic variation within peer networks and how they are using local varieties during face-to-face interactions, in particular with friends. Although stylistic variation and accommodation to the interlocutor have been well described in adults, their emergence and development during childhood remain poorly documented.

Language is fundamentally variable, not only within the speech community but also in the speech of an individual; this intraspeaker variation related to social context is referred to as stylistic variation (Labov, 1972b; Coupland, 2007). The frequency with which an adult speaker uses standard and non-standard variants depends on the social context of the exchange influencing the stylistic choices of the speaker (Chambers et al., 2002). These contextual factors range from the situational context of speech (e.g., formal vs. casual) to the topic of conversation and the identity of the addressee (Labov, 1972b; Rickford and McNair-Knox, 1994). Notably, speakers can modify their linguistic uses to match those of their interlocutors during exchanges. In one of the most comprehensive studies on speakers' accommodation in a natural setting, Coupland (1980, 1984, 2007) recorded an assistant in a travel agency in conversation with a wide socioeconomic range of clients and found that the assistant used more non-standard variants with the clients from the socioeconomic group with the greatest tendency to use these variants. Style-shifting has been also documented when comparing conversations with a friend vs. with an interviewer: Speakers use more vernacular varieties with a friend (Russell, 1982; see also Bell, 1984 for a review of past studies testifying a consistent effect of peer vs. interviewer as addressee in different languages). Adult speakers can also modify their speech in relation to the perceived geographical characteristics of the audience. For example, by studying newscasters on different radio stations in New Zealand, Bell (1984, 2001) showed that they modified their uses of linguistic variants according to the program's audience (national or regional).

A review of the literature on first dialect acquisition spanning the past 40 years delineates the earliest age at which adult-like patterns have been observed for phonological variables, with stylistic adjustments being evidenced as young as 3 years old for some variables (Nardy et al., 2013). The situational context of speech, the addressee, and the topic, all modulate children's use of standard variants (Patterson, 1992: American English in 4, 6, and 8 year-olds). In particular, children use non-standard or local variants more frequently in informal than in formal situations (Díaz-Campos, 2005: Venezuelan Spanish in 3;6–5;11 year-olds) with another child than with an adult (Roberts, 1997: American English in 3;2–4;11) and in routine and play activities than in educational and discipline-oriented exchanges with their mother (Smith et al., 2007: Scottish English in 2;10–3;6 year-olds). These findings lead to the conclusion that the first manifestations of adult-like stylistic patterns of variation emerge at a very young age. Despite a number of studies of stylistic variation in children, the literature review highlights the absence of studies before early adolescence on children's adjustments in their language in relation to peer networks and the identity of their peers.

Therefore, the present study investigated how school-age children use a regional variant within peer networks and modify their speech in relation to friends' geographical background (native or not) and friendship duration (knowing each other since early childhood or not). We focused on a regional variant that is a remnant of the Francoprovençal language the use of which by adults is strongly associated with local identity in rural areas of the French Alps. A previous case-study in the same local area of a native 10-year-old boy indicated that the child differentiated his linguistic uses with his parents, siblings and four friends chosen for their contrasted identity (Martin et al., 2010). The child used the regional variant more with his parents and his long-standing friends. Indeed, friendship duration had a significant influence regardless the friends' nativeness, whereas nativeness was influential for short-term friends but not for long-standing friends. Different stylistic patterns were found for linguistic variables of general French: The child produced fewer non-standard variants with his parents than with other children, whether siblings or friends, but no variation was found in relation to friends' identity. The use of these two categories of linguistic variables (regional vs. general) appears valid to disentangle the social significance of style-shifting in relation to local identity. This case-study constituted a first step for understanding children's regional uses within family and peer networks, but left open a number of issues regarding the relative influence of friends' nativeness and length of acquaintance as well as the possible influences of target child gender on dialectal uses and accommodation with friends. To address these issues, we studied how 10–11 year-old children of both sexes, native of several villages in the same area of the French Alps, used regional and general variants of French with same-age and same-sex friends with contrasted social identity (i.e., nativeness and length of acquaintance). The children were recorded during spontaneous dyadic conversations during free play at school ensuring ecological validity of the situational context of peer speech while also controlling rigorously the characteristics of friends selected in the children's peer network (i.e., age, sex, place of birth, closeness and duration of the friendship).

## Materials and methods

### Participants

All the children lived in the same restricted rural area in four adjoining mountain villages in the department of Haute-Savoie located in the northern part of the French Alps. The children, aged 10–11 years, were recruited from the school of each village. The empirical investigation used to select the target native children and to choose friends who differed only in terms of two criteria—nativeness and duration of the relationship, was carried out in two steps.

First, an exhaustive census of the children attending the two last grades of elementary school was carried out in three of the local schools in order to collect information on children's demographic characteristics and friend networks (from September to December 2007). A fourth school was included a few months later. After receiving informed consent from school headmasters and parents, the children (*n* = 117, from the 6 classrooms of the 4 schools) filled a questionnaire comprising children' and families' demographic information with particular attention to their geographical background (i.e., where they were born and where they lived) and children's friends' identity. To identify children's friends, we used peer nominations, a classic sociometric tool that has proven its validity in the study of school-age children's peer relationships (Barbu, 2003; Cillessen, 2009). To this end, the children had to name their best friend, good friends as well as more peripheral other friends and to indicate whether they were in the same school and classroom and for how many years they had known each other. Questionnaires were cross-checked and completed with schools' registers when necessary.

Second, the first selection criterion to choose the target children was that they were native, that is born and having always lived in the villages or nearby with at least one parent, if not both, who were also native. The second criterion was that the children had friends with contrasted identity in their local peer network. The friends we chose to record during dyadic conversations with the target children had to be of the same age and same sex and to be reciprocal close friends (i.e., the children named each other as best or good friends). Keeping these characteristics constant, we selected friends who presented the maximum contrast for two traits: length of the relationship (i.e., number of years they had known each other) and nativeness. Native friends were defined as described above; non-native children were defined as those who had not been born locally and who had spent part of their life elsewhere. Hence, we were able to select interlocutors from three categories: native friends known for a long time (noted in the following: NL), non-native friends known for a long time (NNL), and non-native friends known for a short time (NNS). Contrary to a previous case-study in the same area (Martin et al., 2010), it was not possible to constitute a category of dyads with native friends known for a short time. This case occurred actually too rarely in the local peer network: when friends are native, they have known each other since early childhood. As the children's friend networks were dense and small, another selection criterion was to avoid as much as possible overlap between the target children's partners. In agreement with the school staffs, children with school difficulties were not included in the pool of target children in order to limit absences during repeated observation sessions. Finally, two target children and their partners were discarded from the analyses after observations and transcriptions: one because of low occurrences of the sociolinguistic variable; the other because of lack of contrast between partners' identity.

Following these selection criteria, the final sample of target children consisted of 13 native children (7 girls: mean age ± SD = 10.4 years ± 0.5; 6 boys: 10.7 years ± 0.5). Their parents (age-range = 37–50 years) worked in the villages or nearby: the fathers were farmers, artisans/craftsmen or worked for the ski tourism; the mothers were farmers or employees in the service/tertiary sector. All the target children, except one, had siblings.

The target children were recorded with each of their three friends with contrasting identities, yielding a total of 39 dyads (Table 1 for a summary of dyads' characteristics, see also Supplementary Table 1 for details), that is a native friend known for a long time (NL: mean length of the relationship ± SD = 7.6 years ± 0.5), a non-native friend known for a long time (NNL: 7.1 years ± 1.0), and a non-native friend known for a short time (NNS: 2.3 years ± 1.1). Long-term friends, whether native or non-native, did not differ in the number of years of knowing (Wilcoxon, *p* > 0.10). Girls and boys did not differ in the duration of the acquaintance with their partners whatever the friends' category (Mann-Whitney, all *p* > 0.20).

**Table 1:** **Summary of the dyads' characteristics: the target children and their three categories of partners**.

Target children = native children from the villages 6 boys and 7 girls, aged 10.5 ± 0.5 years		
NL	NNL	NNS
= with a native friend known for a long time (for 7.6 ± 0.5 years)	= with a non-native friend known for a long time (for 7.1 ± 1.0 years)	= with a non-native friend known for a short time (for 2.3 ± 1.1 years)
Partners = all same-age and same-sex close friends		

### Observational procedure and data collection

All the audio recordings were made in the children's respective schools in a room provided by the teachers at various moments of the day according to the school. Recordings with friends were made during one-to-one interactions between the target children and each of their three selected friends during free play activities with provided play materials (Geomags, puzzles, dominoes…). To ensure spontaneity in peer conversations, no fieldworker was present during the recordings. The children carried a small backpack containing an audio recorder (a mini-disc SONY MZ-RH1, MZ-NH600, or MZ-R70 or an iPod nano) connected to a Lavalier microphone attached to their sweater (SONY ECM-CS10). This recording system facilitated the mobility of the children and promoted natural interactions without requiring the presence of an investigator. Recordings were made between January and June 2008 for three schools and during January 2009 for the fourth school.

The verbal exchanges were transcribed in full using orthographic transcription for all the utterances except for the sociolinguistic variants which were transcribed phonetically. All the productions addressed by the target children to their friend, and by the friends to the target children, were including in the analyses, focusing on conversations, that is, excluding reading, story-telling, singing, sentence-repetitions or self-directed speech. Our analyses were based on a total of 46.9 h of recordings (which corresponds to a total of 38 454 utterances and 291 165 words) with similar total durations of recordings for the three categories of dyads (NL: 16.8 h, NNL: 15.3 h, NNS: 14.8 h) (see Supplementary Table 1 for detailed information per dyad).

### Sociolinguistic variables

We focused on two sociolinguistic variables. The first one is a local variable: the production of the French clitic pronoun as a *y* /i/ rather than as a *le* “him/it,” *la* “her/it” or *les* “them”—for example, “Comment tu *y* sais?” instead of “Comment tu *le* sais” (“How do you know?”), “Elle *y* appelle des aimants” instead of “Elle *les* appelle des aimants” (“She calls them magnets”). We noted this variable (Y) in the following text. The variant *y* is a remnant of Francoprovençal (Tuaillon, 1983), a language in which the singular pronoun placed in front of a verb as a direct object has three forms: masculine, feminine and neuter. The first generation of Francoprovençal speakers to speak French conserved a three-gender pronoun structure rather than adopting the two-gender system of French (masculine and feminine). Because the French language does not have a neutral gender, they used the variant *y* of regional French to refer to inanimate objects and more generally to leave the referent undetermined with regard to gender and numerosity (Fougères and Candea, 2011). The sociolinguistic evaluation of this variant in a small town located 30 kilometers south of our study area showed that most speakers are aware that the variant *y* is not used throughout the French language area and that it is non-standard (Châtellain, 2004). Moreover, speakers with higher socioeconomic status who live outside the area where the variant is used, regard it as a stereotype (i.e., it is stigmatized and identified with the region). Therefore, the variation observed in the variable (Y) by both adults and children seems to be related mainly to geographical criteria and regional identity more than to traditional sociological categories (Martin et al., 2010; Fougères and Candea, 2011).

The second variable is a well-known sociolinguistic variable of general French, namely consisting of variants that are found throughout the French language area: the variable liaison. Liaison is a frequent phonological alternation in spoken French. A liaison consonant—/n/, /z/ or /t/ in the majority of cases (Boë and Tubach, 1992)—appears between two words when the first word is a liaison trigger and the second word begins with a vowel. By observing 100 French speakers from different geographical areas and social backgrounds, Durand and Lyche (2008) established that in some linguistic contexts—such as after an adjective, after a plural noun, after a verb or an invariable word (preposition, adverb, conjunction), the production of this phonological alternation is variable. For example, between an adjective and a noun, a liaison consonant may or may not be produced by adult speakers: “gros éléphant” (“big elephant”) is pronounced either [gʁozelefã] with a /z/ liaison or [gʁoelefã] without any liaison. We named this variable (VL).

Variable liaison is known to be a regularly stratified sociolinguistic variable in adults. A number of studies have shown that the use of the standard variant, i.e., the realization of the liaison, varies with speech style, its production rates being higher in formal situations (Ågren, 1973; Lucci, 1983; Booij and De Jong, 1987; Moisset, 2000), as well as with the speakers' sociodemographic characteristics. Notably, speakers with high socioeconomic status and older speakers realize more variable liaisons than do people with lower socioeconomic status or young speakers (Ashby, 1981; Booij and De Jong, 1987; De Jong, 1991, 1994; Moisset, 2000). The influence of gender is unclear: Some studies reported that women produce more variable liaisons than men (De Jong, 1991, 1994), whereas others found the reverse (Ashby, 1981; Green and Hintze, 1990) or no difference (Moisset, 2000). In children, differences related to family socioeconomic status appear gradually across the preschool years, becoming significant as early as 5–6 years (Chevrot et al., 2011; Barbu et al., 2013). Stylistic variation has been reported: preschoolers use more standard variants with an unfamiliar adult than with peers (Nardy, 2008). Therefore, variation observed in the variable (VL) in both adults and children seems to be related mainly to traditional sociological categories and careful speech style more than to geographical criteria and regional identity.

### Measures and statistical analyses

We calculated individual proportional scores of non-standard variants for each sociolinguistic variable, that is the percentages of the variant *y* for the local morphosyntactic variable (Y) and the percentages of non-realized liaisons for the general variable (VL), produced by the target children addressing each of their three interlocutors and produced by the interlocutors addressing the target children. Children's productions of non-standard variants were analyzed separately for the target children and their friends using generalized linear mixed models in order to evaluate the effects of friends' identity (three modalities: NL, NNL, NNS) and child sex (male, female) as well as their interactions. As measures were repeated, we used glmer analyses with friend identity and child sex as fixed factors and children as a random factor (Kuznetsova et al., 2014). Analyses were performed using R Software (R Core Team, 2014) with the significance level set at *p* = 0.05. Data are represented as means ± standard error of the mean (s.e.m.).

## Results

### Children's productions of the regional variant of (Y)

**Figure 1:**
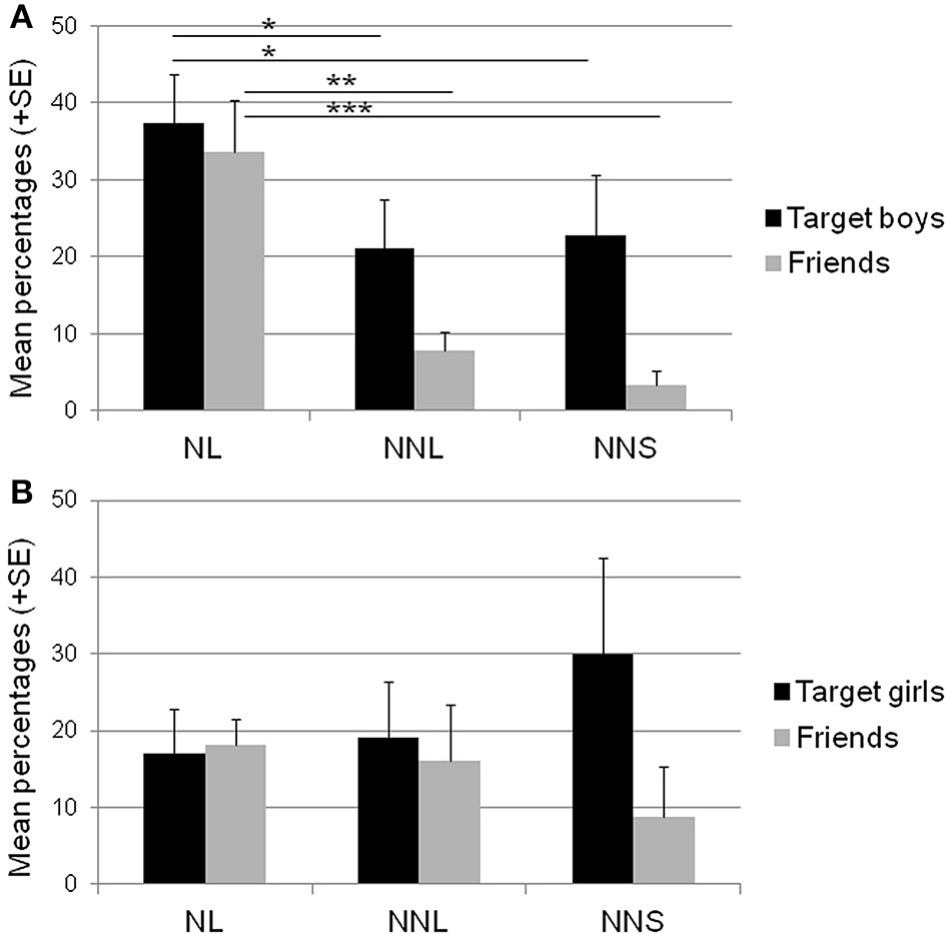
**Children's productions of the regional variant. (A)**: target boys and their same-sex friends, and **(B)**: target girls and their same-sex friends, for the three categories of friends. NL, Native friends known for a long time; NNL, Non-native friends known for a long time; NNS, Non-native friends known for a short time. Bars and error bars represent mean + standard error of the percentages of the non-standard variant of the variable (Y). Glmer post hoc comparisons: ^*^p < 0.05, ^**^p < 0.01, ^***^p < 0.001.

Average scores of the regional variant of (Y) produced by the target children and their three categories of interlocutors (NL, a native friend known for a long time; NNL, a non-native friend known for a long time; and NNS, a non-native friend known for a short time) are shown in Table 2 and Figure 1.

**Table 2:** **Production of the regional variant of (Y) by the target children and their three categories of friends (mean percentages, standard errors in brackets)**.

	**NL**		**NNL**		**NNS**	
	**Targets**	**Friends**	**Targets**	**Friends**	**Targets**	**Friends**
Girls	17.0	18.1	19.1	16.0	30.0	8.7
	(5.9)	(3.4)	(7.3)	(7.3)	(12.5)	(6.6)
Boys	37.3	33.6	21.1	7.9	22.8	3.4
	(6.4)	(6.7)	(6.4)	(2.4)	(7.9)	(1.8)
Overall	26.4	25.3	20.0	12.3	26.7	6.3
	(5.1)	(4.1)	(4.7)	(4.1)	(7.4)	(3.6)

#### Friends: does usage vary in relation to their identity?

The analyses revealed a significant effect of children's identity (i.e., to be a NL, NNL or NNS) [*F*_(2,38)_ = 7.88, *p* = 0.003], but could evidence no significant effect of child gender [*F*_(1, 38)_ = 0.02, *p* > 0.80]. Nevertheless, interaction between child identity and gender approximated significance level [*F*_(2, 38)_ = 3.19, *p* = 0.061], indicating that these two factors should not be considered separately from each other.

The analyses revealed a significant effect of children's regional identity for boys, but not for girls. Post hoc comparisons showed that male friends differed significantly in the production of the regional variant in relation to their local identity (Table 2, Figure 1A): All native friends known for a long time used significantly more frequently the regional variant (33.6% ± 6.7, scores range = 20–65%) than non-native friends known for a long (NL/NNL: *p* = 0.002) or a short time (NL/NNS: *p* < 0.001). No significant differences were found between non-native male friends whatever the length of their relationship with the target children (NNL/NNS: *p* > 0.50). In fact, the production of the regional variant by male non-native friends was not frequent (NNL: 7.9% ± 2.4, score range = 0–17.4%; NNS: 3.4% ± 1.8, score range = 0–10.3%).

Conversely, no significant differences were found between female friends in relation to their identity (NL: 18.1% ± 3.4, NNL: 16.0% ± 7.3, NNS: 8.7% ± 6.6, all *p* > 0.15) (Table 2, Figure 1B). Although most female non-native friends had, as male non-native friends, very low scores (≤10%), a few of them used the variant *y* quite frequently (30% < y ≤ 50%) (see also Supplementary Table 2 for the details of individual scores).

Moreover, although child gender had no general effect on friends' productions of the regional variant, post hoc comparisons revealed a significant effect of gender for native friends only: Native male friends known for a long time produced the regional variant significantly more frequently than did native female friends known for a long time (Table 2, Figures 1A,B) (for NL, boys: 33.6% ± 6.7, score range = 20–65% vs. girls: 18.1% ± 3.4, score range = 0–25%, *p* = 0.050). This gender-related difference was not found for non-native children whatever the duration of their relationship with the target children (for NNL: *p* > 0.20, for NNS: *p* > 0.40).

#### Target children: do they modify their speech in relation to their friends' identity?

The analyses revealed no significant effect of the interlocutors' identity [*F*_(2, 38)_ = 0.85, *p* > 0.40) nor of the children's gender [*F*_(1, 38)_ = 0.28, *p* > 0.60] on the target children's productions of the regional variant. Interaction between child gender and friend identity was below significance level [*F*_(2, 38)_ = 2.77, *p* = 0.08]. The examination of individual scores showed a clear trend for all target boys except one to present their highest scores when talking to a native friend, whereas no pattern appeared for target girls (see Supplementary Table 2 for the details of individual scores) and this led us to investigate separately the data for boys and girls in more depth.

Analyses limited to the boys showed a significant effect of the interlocutors' identity [*F*_(2, 17)_ = 4.14, *p* = 0.049) (Table 2, Figure 1A): They used the regional variant significantly more frequently when addressing a native friend known for a long time (37.3% ± 6.4, score range = 21.7–65%) than when addressing a non-native friend known for a long time (all boys did, NL/NNL: *p* = 0.03) or for a short time (all boys did, except one, NL/NNS: *p* = 0.04). No significant difference was found between non-native interlocutors according to the duration of the friendship (NNL/NNS: *p* > 0.70). Target boys presented very similar scores when addressing both these types of interlocutors (NNL: 21.1% ± 6.4, score range = 11.1–48.1%; with NNS: 22.8% ± 7.9, score range = 0–54.5%).

The analyses of the data for girls revealed no significant effect of the interlocutors' identity [*F*_(2, 20)_ = 1.06, *p* > 0.30) (Table 2, Figure 1B) (with NL: 17.0% ± 5.9, score range = 0–38%; with NNL: 19.1% ± 7.3, scores range = 0–48.1%; with NNS: 30.0% ± 12.5, score range = 0–86.8%). Variability among target girls was important whatever the friends' identity.

Finally, target boys tended to produce more the regional variant than target girls in the context of conversations with native friends known for a long time (NL: *p* = 0.10): They used non-standard variants twice more frequently than girls (37.3% ± 6.4 vs. 17.0% ± 5.9) (Table 2, Figures 1A,B). This gender-related difference was not significant when addressing non-native friends whatever the duration of the relationship (for NNL: *p* > 0.80, for NNS: *p* > 0.50).

### Children's productions of the non-standard variant of general French (VL)

During spontaneous conversations with peers, the children, both the target children and their friends, produced the non-standard variant of the general French sociolinguistic variable at high rates (>90%), that is that in most cases they did not realize liaisons, whatever the friends' identity or the children's gender (for all factors and interaction, 0.10 < *F* < 2.30, 0.10 < *p* < 0.90) (Table 3).

**Table 3:** **Production of non-standard variant (non-realized liaisons) by the target children and their three categories of friends (mean percentages, standard errors in brackets)**.

	**NL**		**NNL**		**NNS**	
	**Targets**	**Friends**	**Targets**	**Friends**	**Targets**	**Friends**
Girls	93.6	95.9	92.7	94.1	94.3	95.2
	(1.4)	(0.8)	(1.5)	(1.0)	(1.7)	(1.4)
Boys	93.7	92.0	93.3	95.6	95.5	94.8
	(2.2)	(3.2)	(1.1)	(1.5)	(0.9)	(2.1)
Overall	93.6	94.1	93.0	94.8	94.9	95.0
	(1.2)	(1.6)	(0.9)	(0.9)	(1.0)	(1.2)

## Discussion

This study investigated the use by native children of the dialectal variants of their local community in peer networks and more especially children's style shifting in relation to their friends' local vs. non-local identity. By doing this, we aimed to understand how children and their peers contribute to the maintenance of a regional dialect. We chose to study this problem here by analyzing the use of a morphosyntactic variable (Y). After describing in depth the friend networks of children who all live in the same rural area of the French Alps, we studied, during free play at school, spontaneous dyadic conversations between the native children and three of their same-age and same-sex best friends who were selected according to their place of birth (native vs. non-native) and their length of acquaintance (since early childhood vs. a few years). Our findings showed that boys, but not girls, modified their dialectal uses in relation to the local identity of their interlocutor: Boys produced the regional variant of (Y) more frequently when talking to native long-standing friends than to non-native friends whatever their length of acquaintance. These adjustments matched their interlocutors' own regional uses as native long-standing friends used more the local variant than did non-native friends. This difference was not evidenced for girls, either for the native target girls or their friends. Not that the girls did not use the local variant, but they showed no clear stylistic pattern and their use presented a great variability ranging from no use at all to high rates whoever the interlocutor. Moreover, a stylistic pattern was evidenced for boys in their use of the regional variant of (Y), but not for the variable of general French, the variable liaison which is a well-known sociolinguistic variable associated with sociological categories of speakers and careful speech, but not with geographical criteria (Durand and Lyche, 2008). Like adults in informal conversations (Ahmad, 1993), children produced the non-standard variant at high rates (i.e., more than 90% of non-realized liaisons). This study reveals that the regional variant was clearly associated with local identity for boys with native male close friends privileging its use during their social interactions.

Our findings confirm the previous case-study conducted in the same geographical area within a boy's family and peer networks (Martin et al., 2010), attesting that 10–11-year-old children are able to use the local dialect subtly and to make adjustments in relation to their interlocutor depending on whether the interlocutor is strongly associated with the local identity (e.g., parents, native friends) or not (e.g., young siblings, non-native friends). The present study also seems to contradict, but in the end clarifies, the influence, reported in the previous case-study, of the duration of acquaintance with non-native friends. As with native friends, the case-study boy used the local variant of (Y) at a high rate with his non-native long-standing friend, as if the length of their acquaintance erased the fact that his interlocutor was not a native of the local community. In the present study, the absence of a category of interlocutors (native friends known for a short time) prevented us from conducting all comparisons as in the case-study to determine fully the effect of the acquaintance duration. Nevertheless, it showed first that this category of friends was in fact not pertinent as it occurred rarely in the actual friend networks of the native children; when children were native and friends, they knew each other since early childhood. Second, although we did not find a difference in boys' productions with non-native friends according to the duration of their relationship, one of the boys presented a pattern similar to that of the case-study boy (see Martin, 2012 for details of individual results). Thus the profile observed in the case study was attested in this larger study, but it was in the minority. Such individual variability in stylistic variation and linguistic accommodation in relation to interlocutor has been already reported in previous studies (Cheshire, 1997, 2009). Taken together, these findings stress the necessity to cross intensive case-studies that are sensitive to the hazards of sampling and more extensive studies. Moreover, although it is important to determine and to understand the general trends of children's dialectal uses and their developmental dynamics, it would be interesting to understand individual variability and its causes as well.

Our main result, evidence of gender differences in children's stylistic adjustments and uses of non-standard variants of a local community, is in line with research concerning adults. A consistent result of several decades of research on phonetic and phonological variation is the linguistic differentiation between men and women, with men using a higher frequency of non-standard forms than women, at least where there is stable sociolinguistic stratification (Labov, 1990, 2002). Indeed when change is in progress, women (often young ones) frequently lead in language change toward non-standard variants (e.g., Eckert, 2000, 2012; Cheshire, 2002). During childhood, seemingly contradictory tendencies have been observed concerning the effects of gender (see Nardy et al., 2013 for a review on phonological variables between ages 2 and 10): 2 of the 11 available studies found that girls used more standard variants, two others found an opposite trend, and seven no difference. Concerning morphological variables, girls were also found to use more standard forms at an early age (Ladegaard and Bleses, 2003). This inconsistency is mainly due to the great heterogeneity of linguistic variables and their social value within a community, subjects' sociodemographic characteristics and situational contexts of speech. For instance in adults, same-sex contexts strengthen both gender differences in sociolinguistic patterns (Takano, 1998) and the degree of convergence between speakers (Pardo, 2006). Our study shows clearly that no useful answer concerning gender differences can be given without reference to the speech context as we did not find overall differences between boys' and girls' dialect uses, but instead we did find differences concerning conversations between native same-sex close friends.

As men show higher uses of local vernacular variants in small rural communities (Holmquist, 1985; Lippi-Green, 1989; Beaulieu and Cichocki, 2002), our study attested similar uses by 10–11 year-old boys living in rural areas in the French Alps. Gender differences have been reported at similar ages for an Italian dialect (Cremona and Bates, 1977) and a rural Syrian dialect (Habib, 2014). The questions of when and how this split between male and female dialect uses occurs during childhood, still remains. The fact that the native boys, but not the girls, adjusted their speech to their interlocutor and privileged the local variant during interactions with same-sex native friends cannot be explained by gender differences in accommodation skills, as previous studies have shown that females generally accommodate more than males (Namy et al., 2002). On the other hand, studies of adults' attitudes toward linguistic varieties indicate that whereas standard variants are associated with social prestige and individual competence, non-standard variants are linked to social skills and solidarity or loyalty toward the native group as well as to virility (Labov, 1972b; Trudgill, 1975). Gender differences may be related to a tendency to view local dialect as more masculine, even in children (Cremona and Bates, 1977). A more satisfactorily explanation may be thus that dialectal uses contribute to the construction of both local and gender identity in relation to an increasing awareness of the social meaning of the local variants during late childhood (Habib, 2014). Nevertheless, although young children do not seem to share widespread adult-like norms, they are not totally devoid of sociolinguistic or gender knowledge as we will see.

An unresolved issue concerns the mechanisms of transmission regarding gender differences in particular. Although many reasons have been put forward to try to explain gender-related differences in sociolinguistic patterns, they have never been satisfactorily accounted for, stressing the complex social construction of gender (Cheshire, 2002; Romaine, 2003). Gender socialization can develop through a great variety of mechanisms, such as parents' differential treatment of girls and boys, social modeling, opportunities to learn through social and physical environment provided to the child, etc. (Leaper, 2002; Bornstein, 2013), some of them have been taken into consideration in sociolinguistic research. For example, English mothers use more vernacular variants with sons than with daughters (Foulkes et al., 2005). Parents also offer children different role models as many of mothers' and fathers' roles and status, including language, traditionally differ. Ladegaard and Bleses (2003) proposed that boys use more non-standard variants, not because of differences in input children are exposed to, but because they model on their same-sex parent or same-sex peers. Nevertheless, gender studies have always failed to prove experimentally that children imitate models of their own sex (Blakemore et al., 2009).

Boys and girls also grow up in different socialization contexts through social network structure. Cross-cultural studies observing parents and children in public places in ten different cultures found that girls were more often in groups with no adult males, whereas boys were more frequently found in all-male groups, and these differences increased with age (Mackey and Day, 1979; Mackey, 1981). Gender segregation is also a pervasive characteristic of peer groups, emerging during early childhood and increasing with age (Barbu et al., 2000). As Labov suggested (2001), dialect formation and change result largely from opportunities for direct social contacts among speakers and are influenced by social relationships between interacting speakers. Strong ties within local social networks both support localized linguistic norms and provide the intensive input to master local linguistic features (Milroy, 2002). Same-sex strong ties among native children within the local network could be an important mechanism accounting for gender differences in dialect transmission and maintenance.

Face-to-face interactions and accommodation between speakers during exchanges, as evidenced among native male friends in the study, play a major role in dialect formation and dynamics on a larger scale (Britain, 2002). Individual short-term accommodation, by leaving traces in memory after exchanges (Pardo, 2006; Delvaux and Soquet, 2007), may become a medium-term convergence among group members (e.g., after a year of daily contact in a peer group at nursery school: Nardy et al., in press; among student roommates after a few months of acquaintance: Pardo et al., 2012) or even long-term convergence that may in turn spread throughout the community at large (Trudgill, 1986). The question at stake is therefore to understand the driving force behind individual short-term accommodation.

Several explanations have been put forward to account for individual short-term accommodation, with strong divergence among authors, opposing high-level psychosocial mechanisms to low-level cognitive mechanisms (Martin et al., 2010). On the one hand, stylistic adaptation has been understood in terms of social signification and motivation (Coupland, 2007), as in the communicative accommodation theory (Giles and Powesland, 1975), audience design (Bell, 1984), acts of identity (Le Page and Tabouret-Keller, 1985) or interactional sociolinguistics (Gumperz, 1982). In this framework, accommodation is a communication strategy by which interacting individuals reduce interpersonal differences or social distance, express solidarity or intimacy, reactivate a shared identity, etc. On the other hand, some authors have argued that short-term accommodation does not result from speakers' overt decisions to coordinate, but instead arises through low-level priming mechanisms (e.g., hearing a linguistic form automatically facilitates production of the same linguistic form), as through interactive alignment of speakers' representations (Garrod and Pickering, 2004) or automatic and unintentional mimesis (Delvaux and Soquet, 2007). However, several reports strongly suggest that automatic priming mechanisms or even mirror neuron systems cannot be the only factors shaping when and how speakers converge (Pardo, 2012 for a review). Instead, situational context of speech like participants' role or identity (Pardo, 2006; Pardo et al., 2013) and individual variability such as speakers' own interpersonal disposition (Horton, 2014) can influence the degree and direction of convergence, whether for phonetic or syntactic features. Understanding the sociocognitive bases of short-term convergence among speakers may be the missing link between psychosocial and cognitive mechanisms, with a main issue that remains to be addressed: when and how social information is encoded and integrated during the linguistic process. Social cognition is an emerging and promising line of research in variationist sociolinguistics, but mostly restricted to date to adult speakers (Campbell-Kibler, 2010); future studies will have to investigate its developmental dynamics.

From a developmental point of view, this unresolved issue questions the early roots of stylistic skills and sociolinguistic knowledge well before individuals are able to elaborate complex communicative strategies to attain identity and relational goals and to show explicit sociolinguistic knowledge. Children actively and progressively construct their linguistic knowledge through their concrete experience in their linguistic and social environment at an early age (Patterson, 1992). In early infancy, children are already able to process environmental regularities, both in linguistic input (Tomasello, 2003) and social roles including gender-typed roles (Eichstedt et al., 2002; Poulin-Dubois et al., 2002; Serbin et al., 2002; Martin and Ruble, 2004; Hill and Flom, 2007). During their preschool years, children become increasingly aware that language variation predicts variation in a range of social groups and can map linguistic information onto social categories (Hirschfeld and Gelman, 1997) including regional dialects (Wagner et al., 2014) and speech styles (Wagner et al., 2010); namely, they become capable of relating different ways of speaking with different categories of speakers and contexts of speech with increasing accuracy. They are also able to adjust their linguistic behavior to social situations (Patterson, 1992; Roberts, 1997; Díaz-Campos, 2005; Smith et al., 2007) and to the social roles they enact in pretend play for instance (Corsaro, 1979; Andersen, 1990; Ervin-Tripp, 2002). Linguistic cues also drive children's social preferences and intergroup attitudes (Patterson and Bigler, 2006; Kinzler et al., 2007), including gender inferences and attitudes (Martin et al., 1995). Thus, young children clearly demonstrate an implicit knowledge of the speech of various categories of speakers and not merely the speech they use to address others; they use this knowledge to adjust their behavior in social situations, interactions and relationships. Nevertheless, still little is known about the sociocognitive process by which children map language variation onto social group differences and situations.

By showing that school-age children are capable of subtle stylistic adjustments with peers in relation to their interlocutors' local vs. non-local identity, our findings contribute to fill gaps in the field of acquisition of sociolinguistic patterns. This research constitutes a significant step in three areas in relation with the use of sociolinguistic variation before adolescence: evidencing stylistic adjustments through accommodation phenomena in relation to peer networks; emphasizing a case of clear-cut gender effects with native male close friends privileging vernacular variants when interacting with each other; and thus understanding how boys contribute to the maintenance of regional varieties. However, this first step needs to be complemented. One of the major issues concerning language variation and change is to determine the linguistic, social and cognitive factors that are involved in the selection and diffusion of variants (Labov, 1994, 2001, 2010). The frequency of sociolinguistic variants also depends on internal linguistic constraints (Labov, 1994). The internal constraints of the variable (Y) remain poorly documented even in adults (Fougères and Candea, 2011) and how linguistic factors affect the production of the regional variant by children remains to be investigated. Future studies will have to consider the developmental dynamics of the variable (Y) in the light of a more thorough analysis of its functioning in adults.

### Conflict of interest statement

The authors declare that the research was conducted in the absence of any commercial or financial relationships that could be construed as a potential conflict of interest.
