# Supplementary material for: The maintenance of regional dialects: a matter of gender? Boys, but not girls, use local varieties in relation to their friends' nativeness and local identity
Source: Front Psychol. 2014 Oct 31;5:1251. doi: 10.3389/fpsyg.2014.01251 (PMC4215785; doi:10.3389/fpsyg.2014.01251)
Supplement: Supplementary file 1 [file Table1.PDF]

Suplementary Table 1. Characteristics of the three types of dyads (target child with a native friend known for a long time: NL, a non-native friend known for a long time: NNL, a non-native friend known for a short-time: NNS) and data samplings: number of years of knowing, number of utterances and words transcribed, and duration (minutes) of audio recordings per dyad. To ensure children's anonymity, names given are pseudonyms.

	Native known for a long time (NL)				Non-native known for a long time (NNL)				Non-native known for a short time (NNS)			
Target children	years of knowing	utterances	words	minutes	years of knowing	utterances	words	minutes	years of knowing	utterances	words	minutes
**Girls**												
Andaine	8	851	5101	62	8	877	5415	65	2	963	5740	68
Célia	8	725	6260	55	5	563	4651	35	1	661	6539	60
Julie	8	851	5101	62	8	888	5867	61	2	667	5805	61
Laurie	7	945	7415	66	7	944	5055	50	4	785	5563	64
Margot	7	1149	7578	85	7	778	5511	56	4	1019	7376	61
Mélina	7	1290	9387	95	7	694	5785	46	1,5	762	7288	47
Zoé	8	1805	11773	124	8	1303	9543	86	3,5	1669	10705	92
Mean	**7.6**	1088	7516	**78**	**7.1**	864	5975	**57**	**2.6**	932	7002	**65**
Total		7616	52615	549		6047	41827	399		6526	49016	453
**Boys**												
Arthur	8	575	4497	55	8	927	6896	88	3	813	7949	65
Brice	7	1550	13065	124	6	1999	14478	118	1,5	1194	9402	92
Dimitri	8	575	4497	55	8	918	7383	62	1	543	3996	41
Lucas	8	523	5115	58	8	412	4652	49	3	963	8304	55
Pierre	7	1550	13065	124	6	1563	10932	124	2	1709	13133	118
Tony	8	667	5463	45	6	1012	7379	77	1	772	7501	62
Mean	**7.7**	907	7617	**77**	**7.0**	1138	8620	**86**	**1.9**	999	8381	**72**
Total		5440	45702	461		6831	51720	518		5994	50285	433
**Overall**												
Mean	**7.6**	1004	7563	78	**7.1**	991	7196	71	**2.3**	963	7639	68
Total		13056	98317	**1010**		12878	93547	**917**		12520	99301	**886**
