# Supplementary material for: The maintenance of regional dialects: a matter of gender? Boys, but not girls, use local varieties in relation to their friends' nativeness and local identity
Source: Front Psychol. 2014 Oct 31;5:1251. doi: 10.3389/fpsyg.2014.01251 (PMC4215785; doi:10.3389/fpsyg.2014.01251)
Supplement: Supplementary file 2 [file Table2.PDF]

Suplementary Table 2. Production of the regional variant (Y) by the target children and their interlocutors for the three categories of friends (**NL**: a native friend known for a long time, **NNL**: a non-native friend known for a long time, and **NNS**: a non-native friend known for a short-time) (in percent). Numbers of occurrences per overall contexts are given in brackets.

	NL		NNL		NNS	
	Targets	Friends	Targets	Friends	Targets	Friends
**Girls**						
Andaine	0 (0/16)	22.2 (2/9)	0 (0/20)	8.7 (2/23)	0 (0/22)	0 (0/34)
Célia	0 (0/12)	25 (6/24)	42.9 (3/7)	10 (1/10)	5 (1/20)	0 (0/28)
Julie	22.2 (2/9)	0 (0/16)	13.8 (4/29)	50 (11/22)	42.9 (3/7)	0 (0/9)
Laurie	14.3 (10/70)	13 (6/46)	19.4 (7/36)	0 (0/55)	12.7 (8/63)	4.5 (1/22)
Margot	9.5 (2/21)	21 (8/38)	9.5 (2/21)	5 (1/20)	5.8 (3/52)	0 (0/44)
Mélina	38 (19/50)	25 (10/40)	48.1 (13/27)	36.4 (8/22)	86.8 (33/38)	47.8 (11/23)
Zoé	35.2 (19/54)	20.5 (15/73)	0 (0/9)	2,2 (1/45)	57.1 (20/35)	8.8 (3/34)
mean ± s.e.	17.0 ± 5.9	18.1 ± 3.4	19.1 ± 7.3	16.0 ± 7.3	30.0 ± 12.5	8.7 ± 6.6
**Boys**						
Arthur	31.4 (11/35)	65 (13/20)	29.1 (16/55)	10 (1/10)	8.6 (5/58)	3.2 (2/63)
Brice	21.7 (18/83)	29 (9/31)	12.7 (10/79)	0 (0/51)	33.8 (22/65)	6.9 (2/29)
Dimitri	65 (13/20)	31.4 (11/35)	48.1 (13/27)	17.4 (4/23)	54.5 (6/11)	0 (0/22)
Lucas	30.4 (7/23)	20 (2/10)	11.1 (2/18)	5.3 (1/19)	0 (0/24)	10.3 (3/29)
Pierre	29 (9/31)	21.7 (18/83)	21 (4/19)	6.1 (2/33)	21.4 (6/28)	0 (0/39)
Tony	46.1 (6/13)	34.5 (10/29)	4.3 (1/23)	8.3 (2/24)	18.2 (2/11)	0 (0/25)
mean ± s.e.	37.3 ± 6.4	33.6 ± 6.7	21.1 ± 6.4	7.9 ± 2.4	22.8 ± 7.9	3.4 ± 1.8
